# Some Aspects of General Peritonitis—II

**Published:** 1908-04-18

**Authors:** 


### Points in Surgery.

### SOME ASPECTS OF GENERAL PERITONITIS.—II.

In the previous article it was said that the best hope of saving cases of general peritonitis lies in adopting the system of continuous rectal irrigation with saline solution; and it was pointed out that several practical difficulties lay in the way of its performance.

Let us go more fully into the treatment. As soon as he comes round from the anæsthetic, the patient is propped up with pillows until he is almost in the sitting position. What is the object of this? It is to prevent infection of the upper half of the peritoneum, if this has not already occurred, and to assist in draining it if it has been involved.

### The Value of Postural Treatment.

From a clinical point of view the peritoneum may be divided, as far as peritonitis is concerned, into two definite portions, which lie respectively above and below the transverse mesocolon. If peritonitis attacks the former of these the outlook is exceedingly grave; and conversely, if only the lower half is attacked, the prospect is comparatively hopeful. If examples were needed to corroborate the truth of this statement, one need only compare the high mortality of perforated gastric and duodenal ulcers (in which the upper half of the peritoneum is necessarily infected) with the comparatively innocuous course of the cases of pelvic inflammation met with in gynæcological practice. In these it is quite the exception to find that the inflammatory condition will not yield to absolute rest in bed combined with hot vaginal douches and fomentations applied to the hypogastrium. It is only rarely necessary to have recourse to operative interference; and then in most cases such interference has for its object the removal of drainage of a residual abscess.

It follows, therefore, that the sitting position is of great value, because it allows the pus or seropurulent material which is always present to make its way towards the pelvis, where its results are far less harmful, and to come to the surface through the drainage tube in the suprapubic wound. In the supine position which is usually adopted the reverse is the case, and we know how fatal are the results. It might be thought that a patient in such a parlous condition is unable to be propped up, or, at any rate, that the condition causes him serious inconvenience. Strangely enough this is not often found to be the case: if it is, morphia may be given to relieve his discomfort.

### The Method of Saline Injection.

But *the* essential part of the treatment consists in the constant injection of warm saline fluid at a temperature of 108° F. into the rectum. To do this a tube is inserted into the anus. There is no need to push it far in. If its extremity lies just inside the sphincter it will be found sufficient. To the tube is attached an irrigating jug or funnel, which is raised about eighteen inches or two feet above the level of the bed. The ideal method is to let in about two ounces of the saline about every ten minutes. As has been already said, this necessitates a nurse in constant attendance, and it is very difficult to keep the fluid at a regular temperature. If it be allowed to cool there is a distinct risk of chilling the patient and doing more harm than good. An easy way of regulating the flow is to have a clip on the tube, and to release it from time to time. If it be found impracticable to keep the fluid at a level temperature, it does nearly as well to inject a pint of fluid or as much as the patient will retain at intervals of two hours. The only disadvantage of this is that the patient if often unable to retain such a large amount at one time, while the more frequent injection of smaller amounts is not likely to give rise to the same difficulty.

In a successful case the fluid is absorbed from the large intestine with the most astonishing rapidity, as much as twenty pints being retained in the first day or two. If this is the case the prognosis is distinctly favourable. The method fails, as might be expected, in those cases in which, owing to the irritable state of the rectum, or from some other cause, the saline is rejected as soon as it is put in.

### Special Advantages of Rectal Infusion.

The saline seems to have a double effect by helping to restore the peritoneum to return to its normal condition and by assisting the heart to overcome the results of the toxæmia; in the latter sense it acts in the same way as does saline infused into a vein. But there seems to be little doubt that when it is absorbed by the intestine its direct action on the peritoneum must also play some part in the process.

Not long ago a series of cases of general peritonitis was treated by frequent infusion of saline fluid into the cellular tissue of the axilla or the buttock after operation. But the experience of those who tried this was that it did not seem to have any effect in reducing the mortality; and we are therefore justified in supposing that the success of the present system depends in some part at any rate on the direct action of the saline on the peritoneum.

So far as it goes, this method of treating general peritonitis by constant injection of saline solution into the rectum appears to be extraordinarily successful; the next obstacle to be overcome is that of discovering some means of inducing patients who reject the saline to retain it. That problem has not yet been solved.

One other supposed difficulty remains to be mentioned; it need hardly be pointed out that one of the most important things in the treatment of peritonitis is to keep the patient's bowels open. Can this be done when the rectum is being constantly irrigated with fluid? The answer is that the fluid is so rapidly absorbed that if its injection be discontinued for half an hour or so the rectum will be found empty, and an ordinary enema can then be given. If purgatives are being administered by the mouth the rectal irrigation will not interfere with their efficiency.

